# Norwegian Translation and Validation of the Pelvic Organ Prolapse/Incontinence Sexual Questionnaire-IUGA Revised (PISQ-IR)

**DOI:** 10.1007/s00192-025-06106-0

**Published:** 2025-03-19

**Authors:** Tone Prøsch-Bilden, Signe Nilssen Stafne, Silje Kristine Sveen Ulven, Susan Saga

**Affiliations:** 1 Norwegian National Advisory Unit on Incontinence and Pelvic Floor Health, University Hospital of North Norway, Tromsø, Norway; 2 Clinic of Rehabilitation, St. Olav’s Hospital, Trondheim University Hospital, Trondheim, Norway; 3 Clinic of Surgery, St. Olav’s Hospital, Trondheim University Hospital, Trondheim, Norway; 4 Department of Public Health and Nursing, Faculty of Medicine and Health Sciences NTNU, Norwegian University of Science and Technology, Trondheim, Norway

**Keywords:** Incontinence, Pelvic floor dysfunction, Pelvic organ prolapse, PISQ-IR, Psychometric validation, Sexual function

## Abstract

**Introduction:**

The Pelvic Organ Prolapse/Incontinence Sexual Questionnaire-IUGA revised (PISQ-IR) measures sexual function in women with pelvic floor dysfunctions (PFD). The aim of this study was to translate the PISQ-IR to Norwegian and to assess its psychometric properties.

**Methods:**

The instrument was translated and reviewed through cognitive interviews with women from the target group and multidisciplinary clinical experts to establish face/content validity and cultural equivalence. Thereafter, a cross-sectional study of women with PFD from two Norwegian University hospitals was conducted. Floor and ceiling effects and internal consistency were calculated for all subscales. Construct validity was assessed through exploratory factor analysis (EFA) and by testing 19 theoretically derived hypotheses.

**Results:**

Of 157 respondents, 111 (71%) women considered themselves sexually active (SA) and 46 (29%) non-sexually active (NSA). Item nonresponse rate varied from 4 to 36% in the subscales. For the NSA subscales, both floor and ceiling effect was detected. EFA mainly supported the original structure for both the SA and NSA subscales, although not completely consistent and with many cross-loadings. Unidimensional factors were assessed and confirmed the presence of one factor within all subscales for SA women and three for NSA women (except NSA-PR). Construct validity confirmed 16 of the 19 predefined hypotheses (84%). All subscales exhibited good internal consistency.

**Conclusions:**

The Norwegian PISQ-IR demonstrated good face/content validity, internal consistency and construct validity, and can be used to assess sexual function among sexually active women with PFD. A small sample size of NSA women precludes drawing firm conclusions regarding structural validity for NSA subscales.

**Supplementary Information:**

The online version contains supplementary material available at 10.1007/s00192-025-06106-0.

## Introduction

Pelvic floor dysfunction (PFD), including urinary incontinence (UI), anal incontinence (AI), and pelvic organ prolapse (POP) is common in adult women. The estimated prevalence is 23–25% of women in general, increasing with age up to 53% among 80-year-olds and older [1, 2]. PFD may have a profound impact on women’s psychological, social, physical, and sexual well-being [3, 4]. In a review of PFD, the prevalence of sexual dysfunction was reported to be 30–50% in the general female population, increasing to 50–83% among women with PFD [5].

The importance of patient-reported outcome measures (PROMs) is widely acknowledged and an essential component in the assessment of outcome after interventions, and its impact on patients’ clinical condition and well-being [6]. The Female Sexual Function Index (FSFI) and Pelvic Organ Prolapse/Urinary Incontinence Sexual Function Questionnaire 12 (PISQ-12) are two common questionnaires previously used to assess the association between PFD and sexual health. Although FSFI has demonstrated good reliability and validity across different populations [7], it is not specific for PFD. Furthermore, PISQ-12 is not validated in a population of women with AI, nor developed within a framework that intentionally included women without a partner, or those who do not consider themselves to be sexually active [8]. To address these limitations, the International Urogynecological Association (IUGA) proposed a revised version of PISQ-12, the Pelvic Organ Prolapse/Incontinence Sexual Questionnaire, IUGA-revised (PISQ-IR) in 2013 [8].

The PISQ-IR was originally validated in > 550 women seeking treatment for UI, AI, and/or POP across the USA and the UK. The questionnaire has demonstrated good evidence for content validity, internal consistency, and some good evidence of test–retest reliability and responsiveness to change [8, 9]. PISQ-IR has been validated in several languages, supporting the internal structure, internal consistency, and criterion validity of the questionnaire [10–16].

In Norway, there are currently no validated questionnaires on sexual function among women with PFD. Although both PISQ-12 and FSFI have been translated into Norwegian, neither of them have been subject to robust psychometric testing, which is important to establish validated measurement instruments. In this study, we therefore aimed to translate the PISQ-IR to Norwegian, and to assess its psychometric properties among women with PFD in the tertiary healthcare system with particular focus on face/content validity, construct validity and internal consistency.

## Materials and Methods

This study required a triangulation of methods involving cognitive interviews of women from the target group, feed-back from clinical experts, and a quantitative survey. The Consensus-based Standards for the Selection of Health Measurement Instruments (COSMIN) guidelines [17] and the PISQ-IR Translation Protocol (IUGA) has guided the work.

### Translation, Pilot-Testing, and Cultural Adaptation

**Fig. 1:**
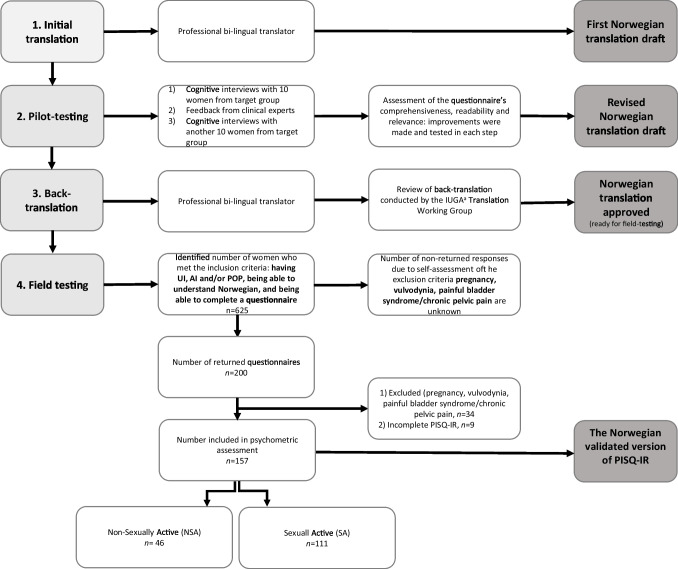
Flowchart of four steps from translation to psychometric testing

The PISQ-IR was translated and cross-culturally adapted to Norwegian through several steps according to the PISQ-IR Translation Protocol (IUGA). First, a translation of the questionnaire was provided by a bilingual translator from English to Norwegian. Second, a pilot-test for comprehensibility, readability, and equivalence was made in three subsequent phases: (1) cognitive interviews with 10 women with PFDs, (2) assessment by multidisciplinary clinical PFD experts, and (3) additional cognitive interviews with another group of 10 women with PFDs following the two preceding steps. Third, another independent bilingual translator back translated the questionnaire from Norwegian to English. Finally, the IUGA Translation Working Group reviewed and approved this version of the questionnaire. Discrepancies were identified and amended between the steps (Fig. 1).

### Study Population and Data Collection

In our study, three groups were recruited. The first group was a sample of 20 women with UI, AI, or POP included in the pilot testing of the questionnaire. This group was recruited from a physiotherapy pelvic floor training class at St. Olav’s Hospital, where they also received information about the study, and volunteered to participate. They were recruited in two rounds with 10 women in each, who underwent cognitive interviews during the pilot testing. They were not enrolled in the subsequent field-testing of the questionnaire. The second group included six clinical experts, comprising a gynecologist, two pelvic floor physiotherapists, two stoma care nurses, and one sexologist, who reviewed and gave feedback on the translated instrument. The correspondence was via email. The cognitive interviews with afflicted women and the evaluation by clinical experts were both part of the pilot-testing of the translated Norwegian version of the questionnaire, and served to establish the foundation for the face/content validity and cultural equivalence between the Norwegian and English versions of the PISQ-IR.

The third group comprised female patients recruited from the Department of urogynecology and gastrointestinal surgery at St. Olavs hospital (Trondheim) and the University hospital of North Norway (Tromsø), from August 2020 to October 2021. This last group was included in the field-testing of the translated PISQ-IR. For inclusion, patients had to be referred to the outpatient clinics diagnosed with UI, AI and/or POP, or any combination of the three, aged 18 years or above, have the ability to understand Norwegian, and have the ability to complete a patient-reported outcome questionnaire. Exclusion criteria included pregnancy or concomitant vulvodynia, painful bladder syndrome or chronic pelvic pain. A predetermined sample of 120 sexually active (SA) and 100 non-sexually active (NSA) women was targeted for inclusion, based on the power calculation derived from the PISQ-IR Translation Protocol. Specifically, performing a factor analysis requires 4–10 respondents per item as a rule of thumb, with 100 respondents as a preferred minimum [18]. In this study, this would require 84–210 SA women (21 items) and 48–120 NSA women (12 items).

In the field-testing, health secretaries identified patients who met the inclusion criteria based on the information given in referrals from General Practitioners. Eligible participants received an information letter and the survey packet together with the invitation to the patients first consultation at the hospital. On the first page of the survey packet, the patients were instructed to tick “yes” or “no” for the exclusion criteria: pregnancy, concomitant vulvodynia, painful bladder syndrome, or chronic pelvic pain (defined as pelvic pain for greater than 6 months). They were further instructed not to fill in the questionnaire or return it if they could tick “yes” for any of these conditions. Respondents anonymously completed the paper-based questionnaire and returned the forms via mail using prepaid envelopes. Responses with a nonresponse rate of > 50% in the PISQ-IR instrument would be excluded after data collection [19].

### Measurements

PISQ-IR consists of two sections, one for non-sexually active (NSA) women and one for sexually active (SA) women, comprising 12 and 21 items respectively (A1). The first core-branching item leads the women to the correct part of the questionnaire, depending on how they consider themselves: NSA or SA, with or without a partner. The two sections are analyzed separately. The NSA section consists of four subscales: condition-specific reasons for sexual inactivity (NSA-CS; Q2c-Q2d-Q2e), partner-related reasons (NSA-PR; Q2a-Q2b), global rating for sexual quality (NSA-GQ; Q4a-Q4b-Q5a-Q6), and condition impact (NSA-CI; Q3-Q5b-Q5c). The SA section consists of six subscales: sexual arousal and orgasm (SA-AO; Q7-Q8a-Q10-Q11), partner-related reasons (SA-PR; Q13-Q14a-Q14b), condition-specific matters (SA-CS; Q8b-Q8c-Q9), global quality (SA-GQ; Q19a-Q19b-Q19c-Q20a), condition impact (SA-CI; Q18-Q20b-Q20c-Q20d), and sexual desire (SA-D; Q15-Q16-Q17). The SA-PR (partner-related) dimension is calculated only if response is given for item 12 (confirmed having a partner). All subscales are analyzed separately and calculation of a summary score is only recommended for SA women [20]. For NSA women, a higher score indicates greater negative impact on sexual function, while for SA women, a higher score indicates less impact and better sexual function.

For hypothesis-testing where PISQ-IR subscales were compared with other existing questionnaires, four related and previously validated self-reported measures were administrated:

*The Female Sexual Function Index (FSFI)* is a 19-item questionnaire and contains the following six subscales: desire, arousal, lubrication, orgasm, satisfaction, and pain. Scores range from 2–36, with higher score indicating greater sexual function [7]. *The St. Mark’s Incontinence score* is a 7-item condition-specific instrument to assess severity of anal incontinence (AI), with a score ranging from 0–24 [21], whereas the *International Consultation on Incontinence Questionnaire Urinary Incontinence Short form (ICIQ-UI SF)* is a 4-item condition-specific instrument to assess severity of UI, with a score ranging from 0–21 [22]. For both the St. Mark’s Incontinence score and the ICIQ-UI SF, higher scores indicate greater severity. *Pelvic Floor Distress Inventory-20 (PFDI-20)* assesses the distress of POP, anorectal and urinary symptoms in three subscales: Pelvic Organ Prolapse Distress Inventory (POPDI-6); Colorectal-Anal Distress Inventory (CRADI-8); and Urinary Distress Inventory (UDI-6) [23]. Each of the three scales is scored from 0 (least distress) to 100 (greatest distress). The sum of the scores of these three scales serves as the overall summary score of the PFDI-20, and ranges from 0 to 300. PFDI-20 and ICIQ-UI SF have been validated in a Norwegian population [24, 25]. In addition, demographic data and past medical history was collected.

### Analysis and Statistics

Statistical analyses were conducted using SPSS version 29.0 (IMB Corp., Armonk, NY). Clinical characteristics between NSA and SA women were assessed using Student’s *t*-test/Mann–Whitney U or chi-square tests. Statistical significance was assumed at *p* < 0.05.

Subscales were scored by mean calculation. According to the recommendation, missing data was handled using listwise deletion, and imputation of missing values was not performed [19]. An item was considered acceptable if the proportion of missing values was less than 15% [18]. Floor and ceiling effects were considered as problematic if more than 15% of respondents achieved the highest- or lowest-possible scores [17]. Internal consistency was examined using Cronbach’s alpha coefficient, where a score > 0.7 was considered reliable [18]. The item-total correlation was examined and items with values below 0.3 were excluded [18].

Construct validity was assessed in two ways: (1) Explorative factor analysis (EFA) assessing PISQ-IR dimensionality, and (2) by hypothesis-testing where PISQ-IR subscales were compared with other existing questionnaires. The dimensionality of PISQ-IR was assessed using explorative factor analysis (EFA). In the EFA, each PISQ-IR subscale was tested individually as unidimensional subscales. In addition, the PISQ-IR multidimensionality was tested by entering all items simultaneously. The suitability of data was confirmed by a significant Bartlett’s test of sphericity, the Kaiser–Meyer–Olkin measure of sampling adequacy exceeding 0.6, and inspection of the correlation matrix identifying correlation coefficients of > 0.3 [26]. Principal component analysis (PCA) with oblique (Promax) rotation was applied to identify item grouping. Kaisers criterion with retention of eigenvalue > 1 and inspection of Cattel’s scree plot guided the number of factors to be extracted [26]. Construct validity was also assessed by 19 theoretically derived hypotheses expressed in terms of expected direction and magnitude of effect (Table 1). For discriminant validity, NSA and SA women were divided into two groups based upon the knowledge that a high burden of symptoms, i.e., the groups defined as St. Marks ≥ 9 [27], ICIQ UI SF ≥ 13 [22], and POPDI-6 > 17 [28], was associated with reduced quality of life and sexual function [5, 29]. Further, in investigation of convergent and discriminant validity, PISQ-IR subscales were compared with subscales measuring conceptually similar constructs, in line with previous publications [10–16]. Nonparametric Spearman rank-sum correlation was used where coefficients were considered low (< 0.3), moderate (0.3–0.59), or high (≥ 0.6 high) [30].

**Table 1:** Construct validity. Confirmation or rejection of hypotheses

Hypothesis tested		Value	Confirmed
Discriminant validity (between known groups)		*p*	
1. The mean score on the subscale “condition impact” is significantly higher for the NSA group with heavy burden of pelvic floor symptoms than for the NSA group with low symptom burden		0.769	No
2. The mean score on the subscale “condition-specific” is significantly higher for the SA patient group with no to moderate incontinence than for the SA group with severe symptoms of incontinence		< 0.001	Yes
Convergent and discriminant validity		Rho	
Correlation expected	Between		
High positive^a^	3. PISQ-IR SA-AO and FSFI-Arousal	0.719	Yes
	4. PISQ-IR SA-AO and FSFI-Orgasm	0.592	No
	5. PISQ-IR SA-D and FSFI-Desire	0.765	Yes
	6. PISQ-IR SA-SUM and FSFI total scores	0.765	Yes
	7. PISQ-IR SA-GQ and FSFI-Satisfaction	0.671	Yes
Moderate positive^b^	8. PISQ-IR SA-PR and FSFI-Satisfaction	0.476	Yes
	9. PISQ-IR NSA-CI and PFDI-20	0.360	Yes
	10. PISQ-IR NSA-CS and PFDI-20	0.336	Yes
Moderate negative	11. PISQ-IR SA-CI and PFDI-20	−0.396	Yes
	12. PISQ-IR NSA-GQ and FSFI-Satisfaction	−0.583	Yes
Low positive^c^	13. PISQ-IR NSA-CS and ICIQ-UI SF	0.281	Yes
	14. PISQ-IR NSA-CS and St. Marks score	0.137	Yes
	15. PISQ-IR NSA-CS and PFDI-20 POPDI-6	0.275	Yes
Low negative	16. PISQ-IR SA-CS and PFDI-20 POPDI-6	−0.164	Yes
	17. PISQ-IR SA-CS and ICIQ-UI SF	−0.405	No
	18. PISQ-IR SA-CS and St. Marks score	−0.186	Yes
Low to no existing	19. PISQ-IR NSA-GQ and FSFI total	−0.073	Yes

^a^ Measure the same construct

^b^ Appear to measure similar but not equivalent constructs

^c^ Do not measure similar construct although greater burden of PFD is associated with deterioration of sexual function

Face and content validity were assessed by a review of cognitive interviews with patients in the target population, and comments/feedback received from clinical experts on a question-by-question basis [18].

### Ethics Approval

Ethical approval was obtained from The Regional Ethical Committees for Medicine and Health (REC) (reference no. 95426/REK sør-øst), the institutional review board at the Department of Urogynecology and Gastrointestinal Surgery at St. Olavs Hospital in Trondheim and the data protection official at both NTNU and the University Hospital of North Norway in Tromsø. Permission to validate PISQ-IR was also granted by the developer of the instrument, IUGA. Written consent was obtained from the women who participated in the cognitive interviews.

## Results

A total of 625 women were approached for the field-test of the study, of whom 200 (32%) returned the survey packets. It was not possible to calculate an exact response rate because we could not determine how many of the 425 non-returned responses were due to self-assessment of the exclusion criteria listed at the beginning of the survey packet. After 34 of the 200 returned questionnaires were excluded due to reporting concomitant pain-conditions, in addition to 9 questionnaires excluded due to incomplete data sets (< 50% of PISQ-IR completed), this resulted in 157 responses available for analysis (Fig. 1). Among these, 46 (29%) women were identified as NSA, and 111 (71%) as SA. UI (82%) was the most common complaint, followed by POP (52%) and AI (52%). The mean age of NSA women was 60 years with 78% being postmenopausal, whereas the mean age of SA women was 51 years with 57% being postmenopausal (*p* < 0.001). The clinical characteristics of NSA and SA women are shown in Table 2.

**Table 2:** Clinical characteristics of the study population

	Non-sexually active *n* = 46		Sexually active *n* = 111		Total *n* = 157		
	*n*	(mean ± SD) [Q1; Q3] or frequency (%)	*n*	(mean ± SD) [Q1; Q3] or frequency (%)	*n*	(mean ± SD) [Q1; Q3] or frequency (%)	*p*
Age (years)	45	60.2 ± 13.3	111	51.3 ± 12.1	111	53.9 ± 13.0	< 0.001
Body mass index (BMI)	44	27.8 ± 5.4	108	26.0 ± 4.1	152	26.5 ± 4.6	0.056
Postmenopausal	46	78.3%	109	47.7%	155	56.8%	< 0.001
Parity	46	2.1 ± 1.0	111	2.3 ± 1.1	111	2.2 (1.1)	0.324
PFD symptom debut	46		109		155		0.012
< 12 months		31%		9%		15%	
1–5 years		41%		43%		43%	
6–10 years		15%		20%		19%	
> 10 years		13%		28%		23%	
Duration of deteriorated sexual function	22		59		81		0.325
< 12 months		23%		22%		22%	
1–5 years		32%		34%		33%	
6–10 years		32%		15%		20%	
> 10 years		14%		29%		25%	
Other diseases^a^	45	36%	109	28%	154	31%	0.383
Previous surgery for PFD							0.071
Hysterectomy and/or BSO	46	28%	108	17%	154	20%	0.125
Prior prolapse surgery	41	29%	102	10%	143	15%	0.009
Prior incontinence surgery	43	23%	106	20%	149	21%	0.660
Previous/ongoing conservative treatment for PFD	43	72%	106	83%	149	80%	0.175
Previously sought professional help for sexual dysfunction	26	0%	109	2%	135	1.5%	NA
ICIQ-UI SF severity levels	45		111		156		0.510
Urinary continent		16%		19%		18%	
Slight UI (1		13%		17%		16%	
Moderate UI		38%		42%		41%	
Sever UI		29%		16%		20%	
Very sever UI		4%		5%		5%	
ICIQ-UI SF score^b^	38	10.6 ± 4.6	90	9.9 ± 4.7		10.8 ± 4.7	0.434
St. Marks severity levels	43		108		151		0.233
Anal continent		37.2		52%		48%	
Moderate AI (1–8)		30.2		20%		23%	
Sever AI (9–24)		32.6		28%		29%	
St. Marks score^c^	33	8.1 ± 5.0	88	7.1 ± 6.1		7.4 ± 5.8	0.149
PFDI-20^b^							
Question 3 (vaginal bulge)	44	66%	111	46%	155	52%	0.025
POPDI-6	46	37.8 ± 26.0	111	27.5 ± 21.5	155	30.5 ± 23.3	0.011
PFDI-20 total score	44	115.5 ± 57.1	111	96 ± 49.8	155	101.5 ± 52.5	0.037
FSFI^d^							
Desire	45	1.2 [1.2; 2.4]	108	3.0 [2.4; 3.6]	153	2.4 [1.2; 3.6]	< 0.001
Arousal	44	0.0 [0.0; 0.5]	110	4.1 [2.7; 5.1]	154	3.0 [0.6; 4.5]	< 0.001
Lubrication	44	0.0 [0;0]	110	5.0 [3.5; 5.7]	154	3.9 [0.0; 5.4]	< 0.001
Orgasm	46	0.0 [0;0]	111	4.4 [3.2; 5.6]	257	3.6 [0.0; 5.2]	< 0.001
Satisfaction	27	2.4 [0.8; 3.2]	105	4.4 [2.8; 5.2]	132	4.0 [2.4; 4.8]	< 0.001
Pain	36	0.0 [0;0]	110	5.4 [2.8; 6.0]	146	4.2 [0.0; 6.0]	< 0.001

*AI* anal incontinence, *BSO* bilateralt salpingo-ophorectomy, *FSFI* Female Sexual Function Index, *ICIQ-UI SF* International Consultation on Incontinence Questionnaire-Urinary Incontinence Short Form, *PFDI-20* Pelvic Floor Dysfunction Index, *POP* Pelvic organ prolapse, *POPDI* Pelvic organ prolapse distress, *St. Marks* St. Marks incontinence score, *UI* urinary incontinence

*p* value is calculated with Student *t*-test/Mann–Whitney U or chi-square

^a^ Diseases that can lead to UI/AI/POP such as diabetes, neurological disease, depression, use of antidepressants, and previous radiation therapy of the pelvis

^b^ Higher scores indicate greater severity

^c^ Higher scores indicate more bother of incontinence. Only score ≥1 are included

^d^ Higher score indicare better sexual functioning

### Face and Content Validity

A first round of cognitive interviews with ten patients with incontinence and/or POP led to moderate amendments in the wording of the Norwegian translation, although the participants experienced the questionnaire as relevant and comprehensive. An important finding was that many participants did not find it easy to choose between the two responses “Not sexually active at all” and “Sexually active with or without a partner” in the core-branch item 1 of the questionnaire: “Which of the following best describes you”. Despite the instruction that being sexually active could be with or without a partner, the interviews demonstrated that many participants were unsure which response to choose. Neither the item nor the responses were changed, since the instructions were considered to be sufficient. In the next round, a multidisciplinary group of clinical experts also considered the content of PISQ-IR as relevant and comprehensive to measure sexual function among women with incontinence and/or POP. However, they suggested moderate amendments in the wording of the questionnaire. This necessitated a second round of cognitive interviews with another ten women from the target group, which however revealed no further need for improvement of the Norwegian translation. By following these steps, a Norwegian version of PISQ-IR with good face and content validity in terms of relevance, comprehensiveness, readability, and equivalence was established.

### Item Nonresponse and Floor and Ceiling Effect

Among the 46 NSA women, 24 (52%) completed all 12 items designated for this group. The nonresponse rates for NSA items varied from 4% (question 6) to 37% (question 2d and 2e). The nonresponse rates for NSA subscales varied from 11% for the global quality rating (NSA-GQ) to 35% for the condition-specific matters (NSA-CS). For the SA women, 81 of the 111 (73%) respondents completed all 21 items. Item nonresponse rates varied from 0% (question 7, 9, 11, 14a-b, 16, 17, 20a) to 11% (question 8b-c). The only SA subscale that had a nonresponse rate > 1% was condition-specific matters (SA-CS) with 10% (Table 3).

**Table 3:** Scale parameters

Scale	No. of items	Missing *n* (%)	Median [Q1; Q3])	Cronbach’s *α* (*n*)	Floor	Ceiling
Non-sexually active (*n* = 46)						
Condition-specific (NSA-CS)	3	16 (35%)	2.3 [1.7; 3.4]	0.68 (25)	20%	10%
Partner-related (NSA-PR)	2	8 (17%)	3.5 [2.4; 4.0]	n.a.^a^	42%	5%
Global quality (NSA-GQ)	4	5 (11%)	3.0 [2.0; 3.9]	0.87 (32)	7%	17%
Condition impact (NSA-CI)	3	7 (15%)	2.7 [1.0; 3.7]	0.87 (36)	15%	31%
Sexually active (*n* = 111)						
Arousal/orgasm (SA-AO)	3	0	3.8 [3.3; 4.0]	0.72 (91)	1%	2%
Partner-related (SA-PR)^b^	3	0	3.3 [3.0; 3.7]	0.81 (98)	1%	21%
Condition-specific (SA-CS)	3	11 (10%)	4.3 [3.7; 5.0]	0.78 (98)	2%	29%
Global quality (SA-GQ)	4	1 (1%)	3.0 [2.3; 3.6]	0.87 (103)	6%	9%
Condition impact (SA-CI	4	1 (1%)	3.3 [2.3; 4.0]	0.92 (109)	8%	25%
Desire (SA-D)	3	0	3.0 [2.3; 3.3]	0.73 (110)	1%	1%

^a^n.a. not assessed (only two items in the scale)

^b^12 women excluded owing to marking off for no sexual partner

Among NSA women, floor effects were seen for the NSA-PR and NSA-CS subscales, with 42% and 20% reporting the worst possible score, respectively. Ceiling effects were seen in NSA-GQ and NSA-CI with 17% and 31% reporting the best possible score. Notably, among NSA women who responded to item 2a (no partner as reason for not being sexually active), 63% reported the worst possible score. Ceiling effects were also seen in the subscales SA-CS, SA-CI, and SA-PR, where 29%, 25%, and 21% reported the best possible score, respectively (Table 3).

### Construct Validity

Twenty-four of the 46 (52%) NSA women completed the 12 items designated for this group, forming the sample for the EFA. The Kaiser–Meyer–Olkin measure of sampling adequacy was 0.68, and Bartlett’s test of sphericity reached statistical significance, supporting the factorability of the correlation matrix. PCA with Promax rotation revealed the presence of four components with eigenvalues exceeding 1, explaining 43%, 14%, 11%, and 9% of the variance, respectively (Table 4). The four factors explained 77% of the variance, revealing differences from the original PISQ-IR structure. Item 5a and 6, originally from the NSA-GQ, were loading on the same factor as the items for NSA-CI. Furthermore, item 2a, originally belonging to NSA-PR, loaded strongest on NSA-GQ with a factor loading of −0.837, although there were cross loadings on both NSA-GQ and NSA-PR (value < 0.4). However, as all items were poorly distributed, interpretating these results is challenging.

**Table 4:** Pattern and structure matrix of four-factor solution for NSA women after PCA with Promax rotation, *n* = 24

	Pattern coefficients				Structure coefficient			
	Factor				Factor			
Item	1	2	3	4	1	2	3	4
Q5a: Frustration	1.055			−0.300	0.909			
Q6: Bothersome	0.911	−0.341			0.764		0.325	
Q5b: Inferior	0.880				0.911	0.380	0.427	
Q5c: Angry	0.795				0.868	0.520	0.321	
Q3: Fear	0.741			0.301	0.851	0.326	0.407	0.483
Q2e: Pain		0.863				0.799		
Q2d: Other health reasons		0.741				0.750		
Q2c: Condition impact	0.382	0.533			0.619	0.672	0.344	0.325
Q2a: No partner	0.314		−0.837	0.376			−0.670	0.342
Q4b: Adequacy			0.804		0.546		0.892	
Q4a: Satisfaction	0.463		0.586		0.675		0.773	
Q2b: No interest				0.899				0.819
Eigenvalue^a^	5.16	1.74	1.35	1.05				
Variance explained	43%	14.5%	11.3%	8.7%				
Total variance explained		57.4%	68.7%	77.4%				

^a^ Eigenvalue refer to the total variance explained by each factor

Of the 111 SA women, 81 (73%) completed all 21 items designated for this group, forming the sample for the EFA. The Kaiser–Meyer–Olkin value was 0.78 and Bartlett’s test of sphericity reached statistical significance. PCA with Promax rotation identified the presence of five components with eigenvalues exceeding 1, explaining 35%, 12%, 11%, 7%, and 6% of the variance, respectively (Table 5). This factor solution explained a total of 69% of the variance. A scree plot revealed no distinct elbow. Promax rotation revealed the presence of a mixed structure compared to the original factor structure [8]. The items from the two factors SA condition impact (SA-CI) and SA condition-specific matter (SA-CS), which conceptually have some similarities, both loaded on one factor (component 1). Component 2 and 3 consisted of the same items as the original SA global quality (SA-GQ) and SA partner related (SA-PR) subscales, respectively. Component 4 consisted of two items from the original SA arousal orgasm (SA-AO) subscale, together with item 16 (frequency desire), which in the original factor structure belongs to the SA desire (SA-D) subscale. Component 5 consisted of two items from the original SA-D subscale, together with item 11 (pain) which originally belongs to the SA-AO subscale. Two items did not load on any component (item 8a) or had factor loadings < 0.4 (item 20a). All remaining variables demonstrated strong loadings (0.465–1.036), and they all load substantially on only one component.

**Table 5:** Pattern and structure matrix of five-factor solution for SA women after PCA with oblique (Promax) rotation, *n* = 81

	Pattern coefficients/matrix					Structure coefficient/matrix				
	Component					Component				
Item	1	2	3	4	5	1	2	3	4	5
Q20d: Angry	0.960					0.904	0.378			
Q20b: Inferior	0.904					0.868	0.358			
Q18: CS fear restrict	0.850					0.840	0.419			
Q8b: Shame	0.749					0.770	0.372		0.350	0.384
Q9: UI/FI with activity	0.745					0.626				
Q8c: Fear	0.663					0.717	0.475		0.389	
Q20c: Embarrassed	0.620					0.760	0.512	0.371		0.458
Q19a: Satisfaction		1.036				0.328	0.920	0.397	0.369	
Q19b: Adequate		1.009				0.332	0.931	0.315	0.461	0.368
Q19c: Confidence		0.873				0.459	0.783		0.358	
Q20a: Frustration		0.343	0.301			0.529	0.691	0.501	0.454	0.493
Q8a: Fulfilled						0.527	0.678	0.339	0.566	0.577
Q14b: Frequency			0.903					0.870		
Q14a: Desire			0.843				0.370	0.868		
Q13: Lack of desire			0.757	0.386	−0.322			0.696		
Q10: Orgasm intensity				0.769			0.311		0.740	
Q16: Frequency desire				0.579			0.564		0.742	0.555
Q7: Sexually aroused			0.319	0.477		0.328	0.520	0.460	0.643	0.404
Q15: Wanting more					0.948					0.751
Q11: Pain					0.584		0.437		0.604	0.642
Q17: Rate desire					0.465	0.426	0.324			0.596
Eigenvalue^a^	7.30	2.61	2.27	1.30	1.1					
Variance explained	34.7%	12.4%	10.8%	6.2%	5.2%					
Total variance explained		47.2%	58.0%	64.2%	69.4%					

^a^ Eigenvalues refer to the total variance explained by each factor

As recommended by the IUGA protocol, unidimensionality was assessed by adding only the proposed items for each subscale separately in the PCA. The one-factor-analysis confirmed the presence of a simple structure within all six subscales for SA women, with factor loadings ranging from 0.489 to 0.939, whereas included items showed strong loadings on only one component. Besides the subscale NSA-PR, which did not meet the criteria for EFA (with only two items included), one-factor analysis also confirmed the presence of a simple structure for NSA women, with strong factor loadings ranging from 0.731–0.931.

Hypothesis testing confirmed 16 out of 19 (84%) predefined hypotheses (Table 1). No significant difference was observed in mean scores on the subscale NSA-CI between the NSA group with high burden of PFD, compared with those with a low symptom burden. Apart from the moderate positive correlation (0.592) between SA arousal and orgasm (SA-AO) and FSFI-orgasm subscales, all other measures of the same construct between PISQ-IR and FSFI provided high positive correlations. Scales measuring similar but not equivalent structures showed moderate correlation in the anticipated direction. Scales measuring unrelated constructs showed low correlation, except from SA condition-specific impact (SA-CS) and ICIQ-UI SF.

### Internal Consistency

Cronbach’s alpha coefficients varied from 0.68 to 0.92, with NSA-CS falling just below the acceptable level of 0.7 (Table 3). Except from the NSA partner related (NSA-PR) subscale, the item-total correlations indicated that all items correlated well with each subscale, with values ranging from 0.44 to 0.84 and 0.37 to 0.88 for the NSA and SA subscales, respectively.

## Discussion

The present study describes the translation and validation of the Norwegian version of the PISQ-IR among women suffering from PFD. In this study, face and content validity and cultural equivalence was established through a meticulous process consisting of reviews from two rounds of cognitive interviews with women from the target group and feedback from a multidisciplinary clinical expert group. Wordings were changed if ambiguity or vagueness was identified during this process. However, some of the participants were unsure whether to respond to “Not sexually active at all” or “Sexually active with or without a partner,” which would classify the respondent as either NSA or SA. The instruction that one could be sexually active both with or without a partner seemed sufficient and no changes were made. In retrospect, an additional instruction about self-stimulation as sexual activity could also have been highlighted, thereby making the distinction between the two easier. Item nonresponse levels were high in NSA subscales. This was also found in Rockwood et al. [19] and several other studies where PISQ-IR have been translated and validated [14–16], and underlines that assessing sexual function is challenging, particularly among women who do not consider themselves as sexually active. Ceiling effect (in terms of best possible score) was found for the subscales NSA-GQ, NSA-CI, SA-CS, SA-CI, and SA-PR, while floor effect (in terms of worst possible score) was found in the NSA-CS and NSA-PR subscales. Similar to our study, both the German and French translation and validation studies of PISQ-IR identified floor and ceiling effects in many subscales [14, 16]. These effects impact the instrument’s discriminative power, making it challenging to detect potential deterioration or improvements after any type of intervention [18]. Despite these limitations found especially in NSA subscales, the Norwegian PISQ-IR can be utilized in both research and clinical practice, but results should be interpreted with caution, keeping these constraints in mind.

The factor structure of PISQ-IR was extracted by Rogers et al. [8] and later confirmed in the Spanish [10] and Chinese versions [11], but most translation and validation studies of PISQ-IR have not assessed the structural validity of the instrument [12–16]. In the Norwegian version, EFA mainly supported the original structure for both SA and NSA subscales, although it was not completely consistent and had many cross loadings. Owing to the small sample size in our study, the results must be interpreted with severe caution. Factors obtained from small data sets do not generalize as well as those derived from lager samples [26]. Therefore, unidimensional factors were assessed and confirmed the presence of one factor within all subscales for SA women and three for NSA women (except NSA-PR).

Furthermore, construct validity was assessed through hypothesis testing of each construct separately, based on assumptions and previous findings of correlations [10–16]. We expected to find moderate to low correlations between PISQ-IR and PFDI-20, St. Marks incontinences score, ICIQ-UI and FSFI due to of the structure of the questionnaires. This is because a condition-specific measurement can describe the impact and bother of symptoms but does not account for sexual function, although greater burden of diseases is associated with lower sexual function [5].

The validity was considered acceptable since 84% (> 75%) of the predefined hypotheses were confirmed [18]. For discriminating validity, we divided NSA and SA women in two groups respectively, based on burden of symptoms. Opposite to what we predicted, the mean score of the subscale “condition impact” was not significantly higher for the NSA women with heavy burden of pelvic floor symptoms compared with NSA women with low symptom burden. This may indicate that the symptom burden does not significantly affect how the condition impacts sexual function in our population.

The single subscales were assessed as constructs, which validly measured the construct to be measured [18], despite the inconclusive nature of the multidimensional aspects of the PISQ-IR. On the basis of Cronbach’s alpha coefficients, all scales demonstrated a good level of internal consistency, in line with other translations of PISQ-IR [10–16]. This reflects that the items in the respective subscales correlated well with each other, thus measuring the same concept [18].

### Strengths and Limitations

This study provides a Norwegian translation and validation of a condition-specific measurement tool for sexual function in women with PFD. A strength of this study is the recruitment of participants from two university hospitals and the inclusion of women across a wide age range which enhances the external validity. In contrast to previous publications [10–16], a notable strength of our study lies in the diversity of PFDs in the study population, particularly the substantial representation of women with AI (52%). This study’s strength also lies in the comprehensive methodology used to validate the PISQ-IR and assess the translation’s psychometric properties, including evaluation of construct validity through 19 predefined hypotheses. The confirmation of 84% of these hypotheses indicates robust construct validity, aligning with theoretical expectations. The findings support the PISQ-IR’s ability to measure sexual function effectively among sexually active women with PFD in a Norwegian context. However, the study has some limitations. The overall sample size in this study is small, particularly the number of women who identified as NSA. We lack information about the women who did not respond to the study, such as whether they were younger or older, or had less or more advanced symptoms. Sexual health and sexual function remain taboo subjects, and some participants may have felt uncomfortable answering the questions. The questionnaire itself was long, and fatigue was observed in answering the PISQ-IR (the last section in the questionnaire) among some of the patients. Scale responsiveness and test–retest reliability is previously established [8, 11, 13, 15, 16]. This was however not assessed, which is a limitation in this study.

### Points for Consideration

By making adjustments that can influence floor and ceiling effect (e.g., highlight self-stimulation as sexual activity in core-branch item 1 and specify that all items need to be answered), the responsiveness of the instrument may be positively affected, making the subscale better equipped to detect change over time. For administration in clinical settings, pre-reversed values can furthermore be an advantage and may make the tool more applicable in daily practice.

## Conclusion

The Norwegian PISQ-IR was found to have good face/content validity, internal consistency and construct validity for each subscale among SA and NSA women, respectively, except for the subscale NSA partner related. Owing to the small sample size, especially among NSA women, conclusions must be considered with caution, and further studies of psychometric properties of the Norwegian PISQ-IR for NSA women is recommended.

## Supplementary Information

Below is the link to the electronic supplementary material.

Supplementary file1 (PDF 724 KB)

## Data Availability

Data supporting the findings of this study are available from the corresponding author upon reasonable request.
