# Supplementary material for: Norwegian Translation and Validation of the Pelvic Organ Prolapse/Incontinence Sexual Questionnaire-IUGA Revised (PISQ-IR)
Source: Int Urogynecol J. 2025 Mar 19;36(7):1435–46. doi: 10.1007/s00192-025-06106-0 (PMC12356724; doi:10.1007/s00192-025-06106-0)
Supplement: Supplementary file 1 — Supplementary file1 (PDF 724 KB) [file 192_2025_6106_MOESM1_ESM.pdf]

PISQ-IR:
Spørreskjema om seksualfunksjon ved fremfall av underlivsorganer, urin- og/eller avføringsinkontinens

I dette spørreskjemaet innebærer seksuell aktivitet, aktivitet alene eller med partner

Spm 1 Hvilken av følgende beskrivelser synes du passer best på deg:

Seksuelt aktiv med eller uten partner	□ →	Gå videre til Spm 7 (del 2)
Ikke seksuelt aktiv i det hele tatt	□ →	Fortsett med Spm 2 (del 1)

Del 1: Spørsmål til personer som ikke er seksuelt aktive

Spm 2 Her er en liste med mulige grunner til at du ikke er seksuelt aktiv. Angi hvor enig eller uenig du er i at dette er grunner til at du ikke er seksuelt aktiv.

		Svært enig	Ganske enig	Ganske uenig	Svært uenig
a.	Ingen partner	□1	□2	□3	□4
b.	Ingen interesse	□1	□2	□3	□4
c.	Har problemer med blære eller tarm (urin- eller avføringsinkontinens) eller fremfall (utbuling eller følelse av utbuling i skjeden)	□1	□2	□3	□4
d.	Fordi jeg har andre helseproblemer	□1	□2	□3	□4
e.	Smerter	□1	□2	□3	□4

Spm 3 Hvor mye har frykten for urin- og/eller avføringslekkasje og/eller utbuling i skjeden (på grunn av fremfall av blære, endetarm eller livmor) å si for at du unngår eller begrenser din seksuelle aktivitet?

□1 Ikke noe å si i det hele tatt
□2 Litt
□3 Noe
□4 Mye

Spm 4 Sett en ring rundt det tallet mellom 1 og 5 som best beskriver hva du mener om sexlivet ditt (sett en ring for hver av punktene a-b).

a.	Fornøyd	1	2	3	4	5	Misfornøyd
b.	Tilfredsstillende	1	2	3	4	5	Utilfredsstillende

Spm 5 Angi hvor enig eller uenig du er i hver av følgende påstander:

		Svært enig	Ganske enig	Ganske uenig	Svært uenig
a.	Jeg føler meg frustrert over sexlivet mitt	□[1]	□[2]	□[3]	□[4]
b.	Jeg føler meg seksuelt utilstrekkelig på grunn av inkontinens og/eller fremfall	□[1]	□[2]	□[3]	□[4]
c.	Jeg føler meg sint på grunn av den betydningen inkontinensen og/eller fremfallet har for sexlivet mitt	□[1]	□[2]	□[3]	□[4]

Spm 6 I det store og hele, hvor vanskelig er det for deg at du ikke er seksuelt aktiv?

□[1] Ikke i det hele tatt

□[2] Litt

□[3] Noe

□[4] Mye

Slutt på spørsmål til personer som ikke er seksuelt aktive

Del 2: Spørsmål til personer som er seksuelt aktive (med eller uten partner)

Resten av spørsmålene i undersøkelsen gjelder et tema som sjelden berøres i slike undersøkelser. Vennligst svar så tydelig og ærlig du kan.

Spm 7 Hvor ofte blir du tent (fysisk opphisset eller kåt) ved seksuell aktivitet?

□1 Aldri
□2 Sjelden
□3 Av og til
□4 Vanligvis
□5 Alltid

Spm 8 Når du er involvert i seksuell aktivitet, hvor ofte føler du deg:

		Aldri	Sjelden	Av og til	Vanligvis	Nesten alltid
a.	Tilfreds	□1	□2	□3	□4	□5
b.	Skamfull	□1	□2	□3	□4	□5
c.	Bekymret/engstelig	□1	□2	□3	□4	□5

Spm 9 Hvor ofte lekker du urin og/eller avføring i forbindelse med ulike typer seksuell aktivitet (med eller uten partner)?

□1 Aldri
□2 Sjelden
□3 Av og til
□4 Vanligvis
□5 Alltid

Spm 10 Sammenlignet med orgasmer du tidligere har hatt, hvor intense er orgasmene dine nå?

□1 Mye mindre intense
□2 Mindre intense
□3 Like intense
□4 Mer intense
□5 Mye mer intense

Spm 11 Hvor ofte har du smerter under samleie? (Hvis du ikke har samleie, setter du et kryss i denne ruten □ og fortsetter med neste spørsmål.)

□1 Aldri
□2 Sjelden
□3 Av og til
□4 Vanligvis
□5 Alltid

Spm 12 Har du en seksualpartner?

1 □ Ja → Fortsett med Spm 13
2 □ Nei → Gå videre til Spm 15

Spm 13 Hvor ofte har partneren din et problem (manglende tenning, begjær, ereksjon osv.) som begrenser den seksuelle aktiviteten for deg?

□[1] Hele tiden

□[2] Mesteparten av tiden

□[3] En del av tiden

□[4] Nesten aldri/Sjelden

Spm 14

	Svært positiv	Ganske positiv	Ganske negativ	Svært negativ
a. Rent generelt, vil du si at **partneren din** har en positiv eller negativ **innvirkning** på ditt seksuelle begjær?	□[1]	□[2]	□[3]	□[4]
b. Rent generelt, vil du si at **partneren din** har en positiv eller negativ innvirkning på **hyppigheten** av din seksuelle aktivitet?	□[1]	□[2]	□[3]	□[4]

Spm 15 Ved seksuell aktivitet, hvor ofte føler du at du vil ha mer?

□[1] Aldri

□[2] Sjelden

□[3] Av og til

□[4] Vanligvis

□[5] Alltid

Spm 16 Hvor ofte har du et seksuelt begjær? Dette kan inkludere at du har lyst på sex, har seksuelle tanker eller fantasier.

□[1] En eller flere ganger daglig

□[2] En eller flere ganger i uken

□[3] En eller flere ganger i måneden

□[4] Mindre enn en gang i måneden

□[5] Aldri

Spm 17 Hvordan vil du rangere ditt seksuelle begjær eller din seksuelle interesse?

□[1] Svært høyt

□[2] Høyt

□[3] Middels

□[4] Lavt

□[5] Svært lavt eller ikke-eksisterende

Spm 18 Hvor mye har frykten for lekkasje av urin, avføring og/eller fremfall (utbuling i skjeden) å si for at du unngår seksuell aktivitet?

□[1] Ikke i det hele tatt

□[2] Litt

□[3] Noe

□[4] Mye

Spm 19 Sett ring rundt det tallet mellom 1 og 5 som best beskriver din opplevelse av sexlivet ditt (sett en ring for hver av punktene a-c).

a.	Fornøyd	1	2	3	4	5	Misfornøyd
b.	Tilfredsstillende	1	2	3	4	5	Utilfredsstillende
c.	Selvsikker	1	2	3	4	5	Ikke selvsikker

Spm 20 Angi hvor enig eller uenig du er i hver av følgende påstander:

		**Svært enig**	**Ganske enig**	**Ganske uenig**	**Svært uenig**
a.	Jeg føler meg frustrert over sexlivet mitt	□[1]	□[2]	□[3]	□[4]
b.	Jeg føler meg seksuelt utilstrekkelig på grunn av inkontinens og/eller fremfall	□[1]	□[2]	□[3]	□[4]
c.	Jeg er flau over sexlivet mitt	□[1]	□[2]	□[3]	□[4]
d.	Jeg føler meg sint på grunn av den betydningen inkontinensen og/eller fremfallet har for sexlivet mitt	□[1]	□[2]	□[3]	□[4]

Takk for at du fylte ut dette skjemaet.
